# Robustness of interstitial photodynamic therapy treatment planning under power and positional uncertainties in light delivery

**DOI:** 10.1038/s41598-026-42421-2

**Published:** 2026-03-05

**Authors:** Shuran Wang, Tina Saeidi, Lothar Lilge, Vaughn Betz

**Affiliations:** 1 Edward S. Rogers Sr. Department of Electrical and Computer Engineering, University of Toronto, 10 King’s College Rd, Toronto, ON M5S3G8 Canada; 2 Department of Medical Biophysics, University of Toronto, 101 College Street, Toronto, ON M5G1L7 Canada; 3 Princess Margaret Cancer Centre, University Health Network, 101 College Street, Toronto, ON M5G1L7 Canada

**Keywords:** Photodynamic therapy, Uncertainty analysis, Light, Simulation, Optimization, Cancer, Engineering, Mathematics and computing, Medical research, Oncology

## Abstract

While computer simulations can accurately model light dose delivery in interstitial photodynamic therapy (iPDT), predicting individual clinical outcomes remains difficult. In addition to biological uncertainties, inaccuracies in light delivery must be considered. Using simulations on virtual brain tumour (glioblastoma) models, we analyze two sources of uncertainty: light source power variations of $\pm 5\%$, $\pm 10\%$, and $\pm 20\%$, and positional deviations during source insertion, modelled as angular errors producing up to $3\textrm{mm}$ displacement. Simulated outcomes show minimal impact from power uncertainty, even at worst-case $\pm 20\%$: the percent difference between maximum and minimum $v_{100}$ does not exceed $9\%$, with tumour coverage only dropping from the targeted $98\%$ to $96.9\%$. Using a new power-uncertainty–aware option in the PDT-SPACE planning tool improves the worst-case minimum coverage from $96.9\%$ to $97.3\%$, eliminating the risk of under-treating. Position uncertainty was simulated by discretizing the space and randomizing source placements, showing a larger negative effect. Power re-optimization on measured post-insertion positions restores tumour coverage to $98\%$, while PDT-SPACE source-position optimization reduces average healthy tissue damage by $36\%$. Combining both yields the most robust performance and minimizes sensitivity to positional deviations, thereby limiting light-delivery errors in iPDT.

## Introduction

Photodynamic therapy (PDT) selectively destroys unwanted tissues by inducing the generation of reactive oxygen species through administered drugs, called photosensitizers, followed by controlled light exposure in tissue in the presence of oxygen. While the biochemical mechanism is well understood^1,2^, the effectiveness of the treatment critically depends on precise knowledge of how light propagates and the resulting photodynamic dose throughout the tissues being treated^3,4^. For superficial PDT applications such as dermatology^5–7^, some superficial applications in head and neck^8,9^ and ophthalmology^10^, direct illumination at the tissue surface allows straightforward computation of the expected light delivery as a function of wavelength, light source output, and effective attenuation. In contrast, for interstitial PDT (iPDT) applications in gastrointestinal^11^, pulmonary^12^, prostate^13^, and intracranial^14^ tissues, light is delivered via optical fibres placed into the tumor through needles or catheters, making treatment outcome highly sensitive to both the light source configuration and the optical properties (OPs) of the tissue (e.g., absorption and scattering coefficients). Modelling the expected light dose throughout the tissues being treated requires sophisticated software simulation, generally via Monte Carlo simulation of photon packets propagating from the light sources throughout the tissue ^15^. Uncertainties in the OPs of the tissue in turn leads to uncertainty in light distribution and treatment outcome; our prior work has simulated, analyzed, and mitigated the impact of these uncertainties in OPs^16,17^. This work, based on glioblastoma multiforme (GBM) brain tumour models, investigates two key practical limitations in light delivery: uncertainty in light source output power and spatial deviations of optical fibres during insertion.

The uncertainty introduced in clinical light power delivery, typically given as optical power per length diffuser [$\textrm{W}\cdot \textrm{cm}^{-1}$], can be kept within $\pm 5\%$ when properly calibrated and traceable to NIST (National Institutes of Standards and Technology) standards^18^. For positional uncertainty, the distance error is typically controlled within the tolerance of $\pm 2 \textrm{mm}$ to $\pm 3 \textrm{mm}$^19,20^. A common strategy to account for this uncertainty is intra-operative (online) dosimetric monitoring based on registered X-ray and ultrasound imaging. Implementing such intra-operative workflows increases surgical duration; Lee *et al.* ^21^ report an average increase of 29.8 minutes in prostate brachytherapy procedures, leading to increased patient discomfort and procedural cost. Our goals are therefore: (1) to predict uncertainties in treatment outcomes arising from power and position variations, and (2) to establish treatment plans that are robust to these uncertainties, reducing the reliance on online dosimetry monitoring.

## Background

The term light dose in this work is defined as the amount of fluence [$\textrm{J}\cdot \textrm{cm}^{-2}$] received at each tissue element, represented by each tetrahedron of the mesh. To evaluate the tissue impact, this fluence is compared to a threshold fluence that is sufficient to kill the tissue represented by the tetrahedron. A tumour is considered under-dosed if the fluence value is lower than the threshold fluence, enabling tumor recurrence and treatment failure; organs at risk (OAR) tissue is considered over-dosed if the fluence value is greater than the threshold fluence, giving rise to therapy-related adverse events and morbidity.

While PDT efficacy depends on multiple clinical and biological factors, delivering an accurate light dose or fluence to the edge of the intended treatment volume is the primary parameter that can be directly controlled and optimized during the intra-operative planning stage. We work to accurately predict the simulated fluence, and use it to generate treatment plans that optimize for both the complete destruction of $>98\%$ of the tumour volume and minimized damage to surrounding healthy tissues, particularly OAR or other eloquent areas. We focus on two controllable variables, source position and power, while assuming others as fixed parameters, including the tissues’ photosensitizer concentrations, OPs, and the sensitivity or responsivity to the PDT-generated cytotoxins. These parameters are assumed to be homogeneous within each type of tissue.

### Light sources in iPDT

In iPDT, light is delivered via fibre-optic diffusers designed to shape the spatial distribution of fluence within tissue. The simplest design is the cut-end fibre, which emits at the distal end and generates a highly localized fluence field. While useful for pre-clinical experimental or highly focal applications, these sources are impractical for treating larger targets^22^. More commonly, cylindrical diffusers are employed, which radiate light radially along a specified fibre length. These can be manufactured with flat (uniform light intensity) or tailored (axially variable light intensity) longitudinal emission profiles, allowing clinicians to better match the illumination to the target volume^23^. Another well-established option is the spherical diffuser, which emits nearly isotropically from a tip. It is advantageous in confined or curved anatomical regions such as cavities, where a uniform radial dose is desirable^24^.

Several other source geometries have been investigated for specific indications. Side-firing and beveled-tip fibres offer directional illumination and have been applied in urologic contexts, such as prostate iPDT^25^. Balloon-catheter diffuser systems are developed to stabilize cylindrical fibres within hollow organs such as the esophagus, blood vessels, or bladder, ensuring reproducible contact and symmetric dose distribution^26,27^. While these variants offer specialized emission patterns, clinical iPDT is primarily performed using cylindrical and spherical diffusers because of their predictable and controllable light distributions. Accordingly, these two geometries form the focus of the present work.

We assume treatment plans are composed of combinations of line and point sources, modelling cylindrical and spherical sources with infinitely small radii, respectively. Modern optical fibres are manufactured with radii commonly less than $0.5 \textrm{mm}$, that is only a small fraction of the typical effective attenuation of light under PDT wavelengths in biological tissues ($\sim 2-5 \textrm{mm}$)^13,28,29^. Thus, the small fibre radius has negligible effect on radial fluence falloff compared to scattering/absorption lengths.

### Open source software tools for iPDT treatment planning

One of the most important factors limiting wider acceptance of iPDT is the lack of robust tools for truly personalized treatment planning: systems that account for patient-specific anatomy and tumour shape, spatially variable photosensitizer uptake and tissue oxygenation, spatially heterogeneous tissue OPs, and procedural uncertainties such as source placement error and power variability — all of which directly alter delivered light dose, safety margins, and thus the expected outcomes^30^. Without such support, treatment decisions rely on heuristics with limited accuracy, adding outcome variability. Extended intra-operative monitoring may be used to compensate, but it often increases procedure time without meaningfully improving the likelihood of therapeutic success.

In this study, we leverage two open-source computer-aided design tools developed in our group to support personalized treatment planning. FullMonte ^15^ computes the light dose and fluence throughout the tissue for a given plan (forward computation), while PDT-SPACE ^31,32^ proposes improved plans using optimization techniques (backward computation).

#### FullMonte: forward simulation of light propagation

Among methods for forward computation of light delivery, Monte Carlo simulations^33^ remain the most established and widely trusted, because they can accurately model photon propagation in heterogeneous tissue volumes. Monte Carlo simulators differ in how tissue anatomy is represented: voxel-based meshes (constructed with small cubes) and tetrahedral or polygonal meshes. MCX^34^ is a voxel-based simulator that provides fast, GPU-accelerated computations and supports cloud and browser access for scalability. SVMC^35^ extends MCX with a hybrid use of voxels and approximation of mesh behaviour at tissue boundaries to improve accuracy. FullMonte^15^, a tetrahedral-based simulator from our group, provides high accuracy due to the tetrahedral geometry and is one of the fastest tetrahedral simulators ^36^, particularly for complex geometries. It can leverage multi-core CPU and GPU resources for efficient computation. Emerging methods include deep-learning-based predictions of Monte Carlo outcomes^37^, which aim to further accelerate forward simulations while maintaining high accuracy.

FullMonte computes light propagation in complex-shaped, turbid tissues using the provided tetrahedral mesh and assigned OPs for each tissue type. The output is the fluence distribution [$\textrm{J}/\textrm{mm}^2$] giving the energy that passes through the surfaces of each tetrahedron throughout the treatment volume. FullMonte is available in multiple configurations: FullMonteSW^15^ runs on multi-core CPUs with multi-threading, while FullMonteCUDA^36^ can leverage CUDA-compatible GPUs for accelerated computation.

#### PDT-SPACE: backward optimization of treatment plan

Prior to PDT-SPACE, works on inverse planning for iPDT were predominantly based on the Cimmino feasibility algorithm^23,38^, which iteratively adjusts source powers to satisfy dose constraints for a fixed source configuration. While robust, these methods focus on constraint satisfaction rather than optimization and do not naturally support source placement optimization or flexible trade-offs between clinical objectives. To provide more personalized and flexible optimizations, our group developed the automated iPDT treatment planning tool PDT-SPACE^17,31,39–41^. The inverse planning problem was broken into two sub-problems: given a certain source placement, assign each source with the optimal power; and optimizing for the source placement itself, utilizing the power optimization sub-problem.

Power optimization with convex optimization An optimization problem requires the formulation of a cost. We utilize a linear program, which is a subset of convex optimization problems^42^.

We follow a general mathematical convention so that the uppercase bold letters ${\textbf {M}}$ represent matrices, the lowercase bold letters ${\textbf {v}}$ represent vectors and the italic letters *x* represent scalars. Elements of matrices or vectors may be represented by labelling indices as subscripts, for example ${\textbf {m}}_i$, $m_{ij}$ or $v_i$.

Let $f({\textbf {x}})$ be the cost function to be minimized^32^, and let **x** be the vector of powers assigned to each source; we formulate the linear program as follows:

1 $$\begin{aligned} \begin{aligned}&\underset{\textbf{x}}{\text {minimize}} & f(\textbf{x}) \\&\text {subject to} & \textbf{A}\textbf{x} \le \textbf{p}_{\text {max}} , \end{aligned} \end{aligned}$$

where $\textbf{A}$ and $\textbf{p}_{\text {max}}$ define the clinician-specified power constraints on combinations of source powers. In our implementation, these constraints are configured such that the total power assigned to all sources does not exceed 1 W to avoid tissue overheating. The cost function $f(\textbf{x})$ is defined as follows:

2 $$\begin{aligned} f_i(\textbf{x}) = {\left\{ \begin{array}{ll} w_i t_i \left( d_{\text {min},i} - \textbf{g}_i \cdot \textbf{x}\right) , & \textbf{g}_i \cdot \textbf{x} < d_{\text {min},i}, \\ w_i t_i \left( \textbf{g}_i \cdot \textbf{x} - d_{\text {max},i}\right) , & \textbf{g}_i \cdot \textbf{x} > d_{\text {max},i}, \\ 0, & \text {otherwise}, \end{array}\right. } \end{aligned}$$

and the total cost is given by

3 $$\begin{aligned} f(\textbf{x}) = \sum _i f_i(\textbf{x}) . \end{aligned}$$

Each tissue element, or tetrahedron, corresponds to one term of the cost function $f({\textbf {x}})$. Each $f_i({\textbf {x}})$ term for tetrahedron *i* is dependent on the tetrahedron’s weight or importance $w_i$, the volume $t_i$, and the amount of over-dosage or under-dosage of fluence at the tetrahedron, depending on the tissue type. The vectors ${\textbf {d}}_{min}$ (for tumour) and ${\textbf {d}}_{max}$ (for OAR) hold the threshold fluence values so the tissue receives its death light dosage; the matrix **G**, where each entry is referred to as $g_{ij}$, stores the fluence values for each tissue element *i*, by simulating only the source *j* with a power of 1W with FullMonte. ${\textbf {g}}_i\cdot {\textbf {x}}$ then gives the expected light dose at each tissue element *i* in fluence [$\mathrm {J/mm}^2$]. This is an application of the superposition of power generated from each light source.

In PDT-SPACE, the ${\textbf {d}}_{max}$ values for certain critical OAR tissues (namely grey and white matters in the brain) have been applied a safety guardband of $90\%$. Therefore, the numbers reported in this work are obtained assuming a death threshold of $0.1\times$ the real death threshold for those OARs.

PDT-SPACE uses the MOSEK^43^ convex optimization tool to solve the defined linear problem, obtaining the optimized source powers ${\textbf {x}}$ [W].

Source location optimization with simulated annealing The simulated annealing (SA) engine^41^ in PDT-SPACE uses the power optimization problem to evaluate and identify better source placements. Starting from a defined initial source placement, or optionally an initial source placement generated using clinical heuristics^31^, SA optimizes for better source locations by repeating the following steps: (1) validate the new placement by checking for any overlaps between sources or any sources placed out of the tumour boundaries; (2) use power optimization to obtain source powers and evaluate cost; (3) evaluate whether to reject the proposed placement with a probability of $1-e^{\Delta {cost}/T}$ when the increase in cost, $\Delta cost$, is positive; (4) perturb the source placements randomly by choosing from a list of move types. The temperature variable, *T*, is given a large value and decreases over time until it reaches a heuristically defined termination value.

## Materials and metrics

### Test geometry

The test geometry is based on a set of segmented 3D tetrahedral brain meshes taken from the Colin27^44,45^ brain atlas; the tumour shapes are approximated by elongated ovoids or several connected spheres to represent clinical GBM images obtained from the cancer imaging archive^46^. The tetrahedral meshes were created and used in the team’s prior works^31,32,40,41^. The tetrahedral meshes (T1−T9) each consist of a total of 423K tetrahedra representing five regions: skull, cerebrospinal fluid (CSF), grey matter, white matter, and tumour. Front views of the tumour models located in the brain are shown in Supplementary Fig. S1, visualized using the Paraview tool^47^. Table 1 gives information on the tumour volume and location, as well as the sources utilized in the source placements used as the baseline for comparison. The light sources are modelled as combinations of infinitely thin line sources that emit light radially along a finite length chosen by PDT-SPACE and infinitely small point sources that emit light isotropically. This is a reasonable model of the thin (1mm diameter) cylindrical diffusers generally used in PDT; these diffusers are commercially available in 5mm length increments^48,49^ and other techniques exist to realize finer gradations in the diffuser length^23,50^.

The initial source placements used in the study were generated by hand based on the heuristic generally utilized in clinical studies and iPDT planning^51–53^. The corresponding source lengths and counts used in each model are shown in Table 1^32,41^. Line sources are placed in a parallel grid fashion, $8-10\textrm{mm}$ from tumour boundaries, separated by a distance of $10\textrm{mm}$, respecting the maximum effective light attenuation of $5\textrm{mm}$^13,28,29^. Point sources are placed in regions that are under-dosed but are closer to tumour boundaries and other sources.

**Table 1:** Tumour model tumour volume, anatomical location, the lengths and counts of light sources used in the initial source placements^41^. Point sources are marked with lengths of 0cm in the table.

Tumour Model	Volume ($cm^3$)	Location	Source Lengths (*cm*) [$\times$Count]
T1	104	Parietal lobe (touch senses)	4 [$\times$6], 3 [$\times$1], 2 [$\times$9], 1 [$\times$3]
T2	110	Cerebellum & brain stem	4 [$\times 6$], 3 [$\times 8$], 2 [$\times 5$], 1 [$\times$2]
T3	33	Occipital lobe (vision center)	1 [$\times 7$]
T4	32	Frontal lobe (motor center)	2.5 [$\times 3$]
T5	22	Frontal lobe (mental functions)	2 [$\times 2$], 0 [$\times 1$]
T6	60	Cerebellum	3 [$\times 6$], 1[$\times 6$], 0 [$\times 2$]
T7	39	Frontal lobe (speech center)	3 [$\times 3$], 1 [$\times 3$], 0 [$\times 1$]
T8	39	Frontal lobe (motor and speech centers)	2 [$\times 6$], 1 [$\times$1]
T9	18	Frontal lobe (emotional area)	2 [$\times 4$]

### Metrics for comparison

We use $v_{100}$ values, i.e., the volume of each tissue receiving $100\%$ its threshold dosage, as the main metric to represent final simulated treatment outcomes. For unwanted tumour tissues, the $v_{100}$ values are represented in percentages, we target the destruction of more than $98\%$ of the tumour region. For OAR regions, the $v_{100}$ values are absolute volumes with units of $\textrm{mm}^3$.

Throughout this work, we define the term *nominal* as the plan or outcome assuming no influence from uncertainties. Therefore, a nominal $v_{100}$ value represents the expected treatment outcome assuming perfect physical delivery of the plan, and serves as the reference for comparison with the $v_{100}$ values simulated with uncertainties.

### Statistical analysis

For comparisons across the nine tumour models, paired t-tests were applied to evaluate differences in tumour and OAR $v_{100}$s between different proposed optimization strategies. P-values $<0.05$ were considered statistically significant. No formal correction for multiple comparisons was applied, given the small sample size and exploratory nature of the study.

### Computation platforms

The algorithms were implemented in C++ and tested on Ubuntu 18.04 machines with 128GB of RAM and 12-core 2.2GHz Xeon E5-2650v4 CPUs; each core can run 2 threads simultaneously for a total of 24 hyper-threads. Monte Carlo simulations are performed with $10^6$ photon packets, repeated runs with different random seeds showed negligible variability in tumour $v_{100}$.

## Power uncertainty

In contemporary clinical practice, optical fibre power uncertainties of approximately $\pm 5\%$ are commonly reported for calibrated PDT delivery systems^18^. To assess robustness against larger uncertainties reported in earlier standards and legacy systems, power uncertainty is analyzed using $\pm 5\%$, $\pm 10\%$ and $\pm 20\%$ ranges.

### Methodology

A worst-case power uncertainty analysis is performed by evaluating treatment outcomes at the maximum and minimum allowable power for each source, representing the upper bounds of possible delivery uncertainty. An alternative would be to model the power output at each source as an independent Gaussian random variable and evaluate the respective treatment outcomes. The worst-case uncertainty model was chosen for a number of reasons. First, it is straightforward to implement. In PDT-SPACE, power optimization is performed using convex optimization based on mathematical formulations. An overly complex uncertainty model will impose unnecessary overhead on the computation. Second, since the delivered light dose scales linearly with source powers, the corresponding worst-case power realizations are expected to reliably bound the minimum and maximum achievable light dose. Therefore, the real outcome of the treatment can be assumed to lie within a safe margin defined by the modelled limits. A more realistic Gaussian-based model may be employed in future work if the extreme bounds prove to be excessively conservative.

For each tumour model, the power optimization feature from PDT-SPACE is performed, fixing the sources at the initial placements described in Test Geometry, to generate the set of nominal powers $\textbf{x}$. The convex optimization procedure assumes each source delivers its nominal (rated) power. In order to apply the worst-case uncertainty model, for each relative uncertainty range $\eta \in \{5\%, 10\%, 20\%\}$, respectively, the program computes the bounds, $x^{max}_i$ and $x^{min}_i$, on the power delivered by a source *i* as a function of the nominal power chosen for that source by PDT-SPACE, $x_i$, using the equations:

4 $$\begin{aligned} \begin{aligned} x^{max}_i&= x_i \cdot (1+\eta ), \\ x^{min}_i&= x_i \cdot (1-\eta ). \end{aligned} \end{aligned}$$

The maximum and minimum bounds are then put into power vectors ${\textbf {x}}^{max}$ and ${\textbf {x}}^{min}$, respectively, to perform FullMonte and obtain two sets of final fluence results. Both sets of final fluence are converted to sets of $v_{100}$ values, which we refer to as $v_{100}^{max}$ and $v_{100}^{min}$, respectively.

### Effects of power uncertainty

We employ the procedure described in Methodology to determine $v_{100}^{max}$ and $v_{100}^{min}$ values for each of the 9 tumour models and computed the geometric averages across all tumour models. Geometric averages are shown since they best capture the underlying trend while reducing the influence of outliers. Table 2 shows the geometric averages for all $v_{100}$ values, and the spread of the $v_{100}$ distributions, defined as $\Delta v_{100}=v_{100}^{max}-v_{100}^{min}$. These values are also expressed in terms of the percentage of relative change, defined as $\Delta v_{100}^\%=\frac{\Delta v_{100}}{v_{100}}\times 100\%$. To give further insight into how much the tumour coverage varies with power uncertainty, we have also listed the absolute maximum and minimum tumour $v_{100}$ values across all simulations and tumour models, as $V_{100}^{{\textbf {max}}}$ and $V_{100}^{{\textbf {min}}}$.

**Table 2:** Geometric averages of $v_{100}$ values across 9 tumour models under source power uncertainty. Reports the upper bounds ($v_{100}^{max}$, $v_{100}^{min}$), the distribution spread ($\Delta v_{100}$), relative change ($\Delta v_{100}^{\%}$), and absolute extrema across all simulations ($V_{100}^{\textbf{max}}$, $V_{100}^{\textbf{min}}$).

	±5% Uncertainty			±10% Uncertainty			±20% Uncertainty		
	Grey Matter (mm^3^)	White Matter (mm^3^)	Tumour (%)	Grey Matter (mm^3^)	White Matter (mm^3^)	Tumour (%)	Grey Matter (mm^3^)	White Matter (mm^3^)	Tumour (%)
${\textbf {v}}_{\textbf {100}}$	65.6	43.8	98.0%	65.6	43.8	98.0%	65.6	43.8	98.0%
${\textbf {v}}_{\textbf {100}}^{\textbf {max}}$	66.3	44.3	98.1%	67.0	44.7	98.1%	68.3	45.5	98.3%
${\textbf {v}}_{\textbf {100}}^{\textbf {min}}$	64.8	43.3	97.6%	64.1	42.8	97.5%	62.4	41.7	97.3%
$\Delta {\textbf {v}}_{\textbf {100}}$	1.50	0.93	0.44%	2.91	1.87	0.61%	5.83	3.79	1.01%
$\Delta {\textbf {v}}_{\textbf {100}}^\%$	2.3%	2.1%		4.4%	4.3%		8.9%	8.7%	
**Max Tumour** $V_{100}^{{\textbf {max}}}$ **Across All**			98.2%			98.4%			98.5%
**Min Tumour** $V_{100}^{{\textbf {min}}}$ **Across All**			97.3%			97.1%			96.9%

From Table 2, we observe that the overall uncertainty in final $v_{100}$ outcomes is small. Even with a $\pm 20\%$ source power uncertainty, tumour tissue destruction varies by only 1% on average over the 9 tumours, and the OAR damage varies by less than 9%. Table 2 also indicates a general tendency for $v_{100}$ uncertainties to scale with the magnitude of source power uncertainties. It can be seen that the minimum tumour coverage in the most extreme case drops faster than the maximum tumour coverage rises, but only down to 96.9% in the $\pm 20\%$ uncertainty model. If the clinician considers this possibility of under-treatment unsafe, PDT-SPACE offers robustness-oriented mitigation strategies, as detailed in the following section.

### Reducing the impact of power uncertainty

In order to reduce the effect of uncertainty in power delivery, we directly modify the cost function $f({\textbf {x}})$ defined for convex optimization over source power ${\textbf {x}}$. Two possible enhancements to PDT-SPACE were proposed and evaluated.

1. The most intuitive approach is to design the cost function to penalize both the over-dosage in OARs and under-dosage in tumours at both extremes of the light source power delivery. Thus, the first cost function formulation, $f_{max+min}({\textbf {x}})$, where ${\textbf {x}}$ is the vector of powers to each light source, is defined as follows to sum both treatment plan costs at the minimum and maximum source powers:
  5 $$\begin{aligned} f_{max+min}({\textbf {x}})=f({\textbf {x}}\times (1+\eta ))+f({\textbf {x}}\times (1-\eta )). \end{aligned}$$
2. As shown above, under-dosage of the tumour due to source power uncertainty is a more significant problem than over-dosage of the OAR. Hence, our second cost function, $f_{min}({\textbf {x}})$, is defined as follows, optimizing power by putting a lower margin on the power to consider the minimum power that could occur due to light source power variation:
  6 $$\begin{aligned} f_{min}({\textbf {x}})=f({\textbf {x}}\times (1-\eta )). \end{aligned}$$

#### Results

**Fig. 1:**
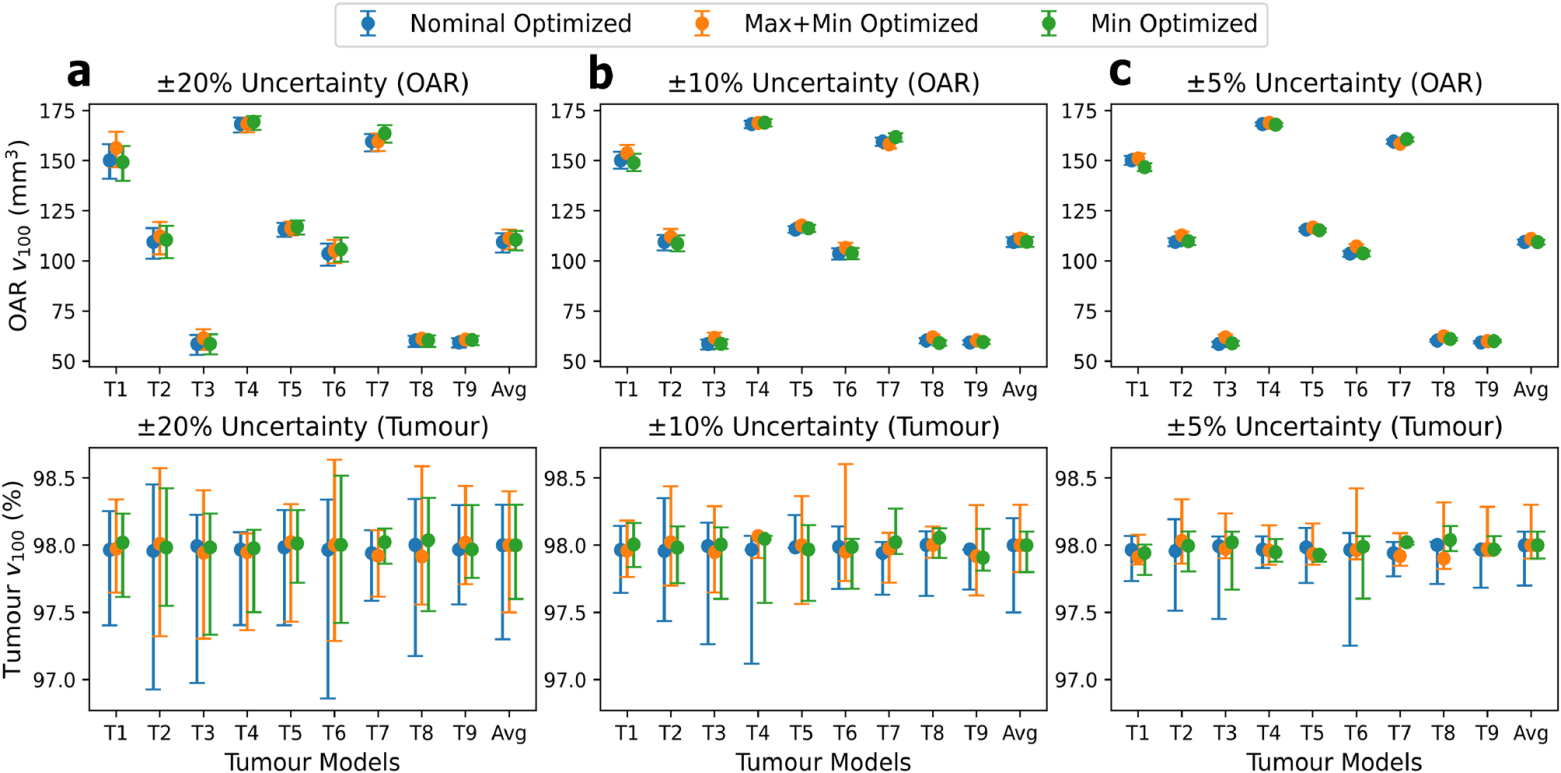
Worst-case $v_{100}$ range comparisons on power uncertainty-aware optimization options. OAR $v_{100}$ sums (top) and tumour $v_{100}$ (bottom) for source power uncertainties ($\eta$) of (a) 20%, (b) 10% and (c) 5%.

We use PDT-SPACE with its original cost function $f(\textbf{x})$ (nominal optimization, assuming no source power uncertainty) and our two modified formulations, $f_{max+min}(\textbf{x})$ and $f_{min}(\textbf{x})$, to select light source powers for the 9 Colin27 virtual brain tumour models, allowing comparison of uncertainty-aware optimizations against the nominal baseline. The $v_{100}$ values for both the sum of OARs and tumours are plotted in Fig. 1. Dots indicate the treatment plan outcome if all light sources exactly match their nominal power, while the whiskers indicate the outcomes at the extremes: either the minimum possible power or the maximum possible power. The geometric averages of the 9 tumour models are shown as the last group in the sub-plots. Table 3 lists detailed numerical comparisons for the geometric average results over the 9 tumours.

**Table 3:** Comparison between Nominal, Max+Min, and Min power optimization options. Reporting geometric average $v_{100}$ values across 9 tumour models under source power uncertainty.

	±20%			±10%			±5%		
	Nom	Max+Min	Min	Nom	Max+Min	Min	Nom	Max+Min	Min
**Grey Matter + White Matter OAR** ($mm^3$)									
$v_{100}$	101.2	103.1	102.2	101.2	103.2	101.1	101.2	103.1	101.3
$v_{100}^{max}$	105.5	107.4	106.7	103.4	105.5	103.5	102.3	104.3	102.4
$v_{100}^{min}$	95.8	97.5	96.8	98.6	100.6	98.6	99.9	102.0	100.1
$\Delta v_{100}$	9.65	9.86	9.92	4.81	4.94	4.81	2.44	2.38	2.36
$\Delta v_{100}^\%$	9.54%	9.57%	9.70%	4.75%	4.79%	4.76%	2.41%	2.31%	2.33%
**Tumour (%)**									
$v_{100}$	98.0	98.0	98.0	98.0	98.0	98.0	98.0	98.0	98.0
$v_{100}^{max}$	98.3	98.4	98.3	98.1	98.3	98.1	98.1	98.2	98.1
$v_{100}^{min}$	97.3	97.5	97.6	97.5	97.7	97.8	97.6	97.9	97.8
$\Delta v_{100}$	1.01	0.92	0.70	0.61	0.55	0.35	0.44	0.36	0.22
**Maximum and Minimum Tumour Coverage Across All Cases**									
$V_{100}^{{\textbf {max}}}$	98.5	98.6	98.5	98.4	98.6	98.2	98.2	98.4	98.1
$V_{100}^{{\textbf {min}}}$	96.9	97.3	97.3	97.1	97.6	97.5	97.3	97.8	97.6

All worst-case outcome metrics for tumour coverage ($v_{100}^{max}$, $v_{100}^{min}$, $V_{100}^{{\textbf {max}}}$, and $V_{100}^{{\textbf {min}}}$) are comparable between the Max+Min and Min optimization strategies. Both uncertainty-aware strategies reduce the risk of tumour under-dosage under extreme power uncertainty, as reflected by improved worst-case $V_{100}^{{\textbf {min}}}$. The Min optimization yields the smallest tumour $v_{100}$ variability, approximately $0.2\%$ lower than Max+Min and $0.3\%$ lower than Nominal. Paired t-tests across the nine tumour models show statistically significant differences in tumour $v_{100}$ between Nominal and Max+Min ($p\approx 0.02$), Nominal and Min ($p\approx 0.0004$), and Max+Min and Min ($p\approx 0.0002$).

For OAR exposure, mean $v_{100}$ increases are observed; however, while Nominal vs. Max+Min and Nominal vs. Min show statistically significant differences ($p<0.05$), differences between Max+Min and Min, as well as differences in OAR variability ($\Delta v_{100}$), are not statistically significant ($p>0.05$).

These results do not match with our initial hypothesis that optimizing solely for minimum power would reduce under-dosage without adequately controlling over-dosage. The observed behaviour is likely due to the additive formulation of the Max+Min objective, which leads to a compromise solution when optimizing simultaneously for both power extremes. In contrast, Min-only optimization is more effective, consistent with the observation that the worst-case tumour coverage generally occurs at the minimum delivered power.

## Position uncertainty

**Fig. 2:**
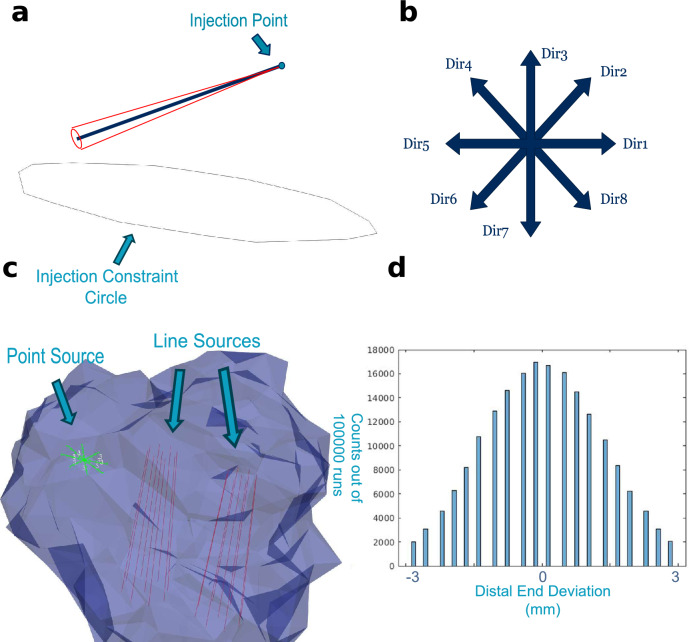
Illustration of position uncertainty for light source injection. (**a**) Conceptual visualization of angular deviation of a needle injection about its injection point. (**b**) The 8 principal directions used to discretize deflection directions, defined on a plane normal to the source axis. (**c**) Visualization in Paraview^47^ of two line sources and one point source inside T5’s tumor volume. Red lines indicate the line sources deflected along the 8 directions by the maximum distance (3mm); green rulers show the deviations from nominal to the 3mm deflected positions for the point source in the 8 directions. The injection constraint is represented by a black circle at the injection point. (**d**) Histogram of angular deviations generated from 100,000 samples. For each sample, a direction is first chosen from the 8 predefined directions, and a deflection magnitude is drawn from a truncated Gaussian distribution centred at 0mm, limited to $\pm 3$mm (samples outside this range are resampled). Positive magnitudes are assigned to Dir $1-4$ and negative magnitudes to Dir $5-8$, producing the discretized distribution shown.

The uncertainty in the physical placement of light sources is assumed to be zero at the point of injection, where points can be accurately identified without internal measurements. Then an angular Gaussian distribution of deviation was modelled about the defined injection point. Since clinical needle- or catheter-based procedures can typically constrain placement uncertainty within approximately $\pm 2\sim 3\textrm{mm}$^19,20^, we choose to be conservative by modelling with the absolute maximum deviation of 3mm. The model evaluates the expected treatment results obtained on the placement where the distal end of the line source (or the point source itself) lies within the circle obtained by rotating the source about the injection point, where all points on the circle are exactly $3\textrm{mm}$ apart from the original end point. See Fig. 2a.

### Implementation and methodology

Since the uncertainty model requires information on injection points, we assume the user always provides an injection constraint^31^. Specifying an injection constraint means defining a surface (described as a circle) through which light sources are allowed to be injected; the injection point is defined as the intersection between the extension of the light source and this surface. We define the injection constraints to closely represent the mesh’s outer surface (the skull in our test cases), while encircling all insertion points for the human-defined initial source placements. This ensures that the deflections we model accurately reflect needle- or catheter-based insertion into tissue.

Geometrically, positional uncertainty is modelled as an angular deflection of each light source about its injection point. The deflection direction is assumed to lie on a circle normal to the source axis, representing all possible angular deviations about the insertion trajectory. The insertion length (from the injection point to the distal end of the source) and the source length (from the proximal to distal end) are held constant, as these quantities can be precisely controlled during the procedure. The maximum angular deviation is determined from an isosceles triangle, where the two equal sides correspond to the insertion length and the base corresponds to a displacement of 3mm at the distal end.

To analyze the impact of source position uncertainty, we evaluate the optimized plan generated by PDT-SPACE on a large number of deviated source positions that are obtained by sampling the source deviation probability distribution. It is impractical to simulate an infinite number of angular deviation possibilities, thus we discretize the sample space. To discretize angular deflection directions, we define 8 equally spaced principal directions (Dir1–Dir8) on the plane containing the injection constraint circle, separated by $45^\circ$ increments, as illustrated in Fig. 2b. The Dir1 direction is defined by obtaining a random vector lying in the defined injection constraint plane^31^. A random variable is used to sample the 8 directions using uniform distribution. Figure 2c shows an example of the sources deviating by their maximum amount of 3 mm, in all 8 principal directions for 2 line sources and 1 point source on the tumour model T5. After the deflection direction is selected, the deviation angle is again discretized into 10 pieces: 0.05, 0.15, ..., 0.95; the numbers are multiplied by the worst-case deviation angle (to approximate the maximum $3\textrm{mm}$ deviation), to obtain the sampled angle. Simulations are performed with the following experimental settings:

1. **Nominal**: The simulated result assuming no position uncertainty exists - baseline of comparison.
2. **Worst case**: Each light source is deflected by the maximum amount (3mm) in a random direction chosen from the 8 predefined directions. This sampling is repeated over multiple iterations to generate a histogram of possible outcomes.
3. **Gaussian**: For each source, a random direction is first selected from the 8 predefined directions. A deflection magnitude is then obtained using the $0.05-0.95$ quantiles to sample a discrete space, scaled to approximate a Gaussian distribution centred at 0mm, with $\sim 95\%$ of samples lying within 3mm. Samples exceeding the range are discarded and resampled. Figure 2 (d) shows the resulting discretized histogram of deflection magnitudes along the 8 directions (Dir $1-4$ counted towards positive, Dir $5-8$ counted towards negative). This procedure ensures that deflections are random in both direction and magnitude while remaining within realistic bounds.

In total, we perform $8 + 1 + 8\times 10 = 89$ FullMonte simulations for each source. Note that the program has no knowledge of the injection point for point sources; thus, it assumes its injection point to be the centre of the injection constraint circle. After performing FullMonte simulations on the source positions, the expected resulting fluence distribution is computed using linear superposition of the individual source contributions, as implemented in the PDT-SPACE power optimization^40^.

### Results

**Fig. 3:**
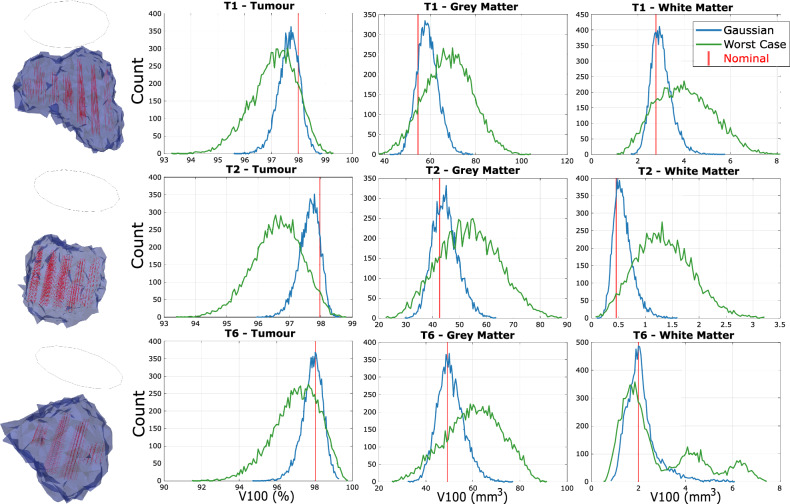
$v_{100}$ histograms under source position uncertainty for the largest 3 tumour models. Blue curves are histograms sampled assuming a Gaussian source position uncertainty distribution; green curves are histograms sampled assuming Worst Case deviations. Nominal $v_{100}$ values assuming 0 uncertainty are shown by vertical red lines. T1 has 19 line sources; T2 has 20 line sources; T6 has 12 line sources and 2 point sources. Tumour shapes, the injection constraint circles, and worst-case light source deflections, are visualized using Paraview^47^.

Figure 3 shows histogram plots of simulated treatment outcomes for the largest 3 tumors over 10,000 random samples under the Gaussian and worst case source position uncertainty assumptions. The horizontal axis shows the $v_{100}$ values, and the vertical axis shows the number of appearances for each small range of $v_{100}$ values within the 10,000 runs. The red vertical lines are the nominal $v_{100}$s. For better visibility, we group the tumour models by source count, based on the data listed in Table 1. T1 and T2 are the largest set of tumour models with $\sim 20$ sources; T6 can also be grouped as one of the larger tumours with 14 sources (Fig. 3). T3, T7 and T8 have $7\sim 8$ sources and are thus grouped in the next tier (Supplementary Fig. S2). T4, T5 and T9 are grouped in the tier with the least number of sources ($3\sim 4$ sources) (Supplementary Fig. S3). The Gaussian distribution shows more realistic expected outcomes, and the worst-case scenario shows the upper-bound overestimation of errors.

In general, the expected values of the $v_{100}$s are slightly worse than the nominal values - OAR damage is higher and tumour destruction is lower. The worst-case deviations from the nominal result are generally $2-3\times$ larger than those of the Gaussian distributions in terms of both expected values and spreads.

### Effect of power re-optimization

This subsection analyzes the impact of re-optimizing PDT-SPACE light source powers after measuring the actual positions of the inserted sources. In practice, once the sources are placed, their deviated locations can be measured and used as input to the optimization algorithm. The re-optimization then chooses new source powers, independent of the original nominal plan, to achieve the prescribed light dose distribution within the tumor. By explicitly accounting for the measured source positions, this step compensates for placement deviations and ensures that the intended dose coverage is maintained. This recalculation step is performed offline using the measured geometry and does not require continuous online dosimetry monitoring.

**Fig. 4:**
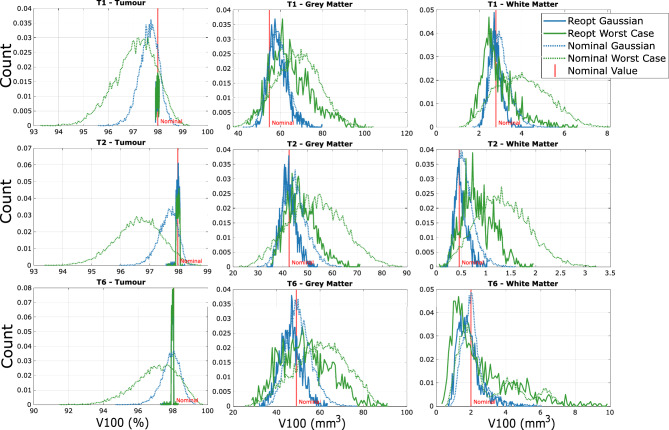
Comparison of $v_{100}$ for the largest three tumor models using nominal-optimized powers (Nominal) versus re-optimized powers (Reopt). Nominal powers are calculated based on the planned (intended) source locations, while re-optimized powers are recalculated after measuring the actual (deviated) source positions. Dotted curves correspond to nominal powers and solid curves correspond to re-optimized powers. Tumor T1 has 19 line sources; T2 has 20 line sources; T6 has 12 line sources and 2 point sources.

Figure 4, Supplementary Figs. S4 and S5 show the comparisons of histograms between outcomes following PDT-SPACE optimization, assuming planned (intended) source locations, vs. when PDT-SPACE re-optimizes the source power choices using the actual (deviated) source locations.

The nominal power optimization curves are obtained using 10,000 samples, and the re-optimized power curves are obtained using 1,000 samples. Performing power re-optimization is computationally expensive; therefore, a smaller number of samples was used for this analysis, which was sufficient to obtain stable histogram estimates. Both histograms are normalized to percentage counts to enable fair comparison.

We can see that the larger tumour models in Fig. 4 show a consistent improvement with the use of power re-optimization — the tumour $v_{100}$s are constrained at $98\%$ since the program optimizes targeting that exact coverage; the OAR $v_{100}$s have smaller spreads, and the expected values are reduced as desired. When we look at the smaller tumour models, while the tumour $v_{100}$s are still controlled at $98\%$, the OAR damages typically increase slightly to compensate for the constraint on the tumour $v_{100}$. These observations are supported by paired t-tests across the nine tumour models, which show statistically significant improvements in tumour coverage with re-optimized power ($p\approx 0.02<0.05$), while OAR differences are mostly not significant ($p\approx 0.26>0.05$).

**Fig. 5:**
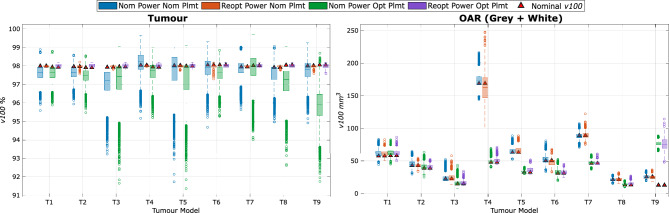
Complete Gaussian sampled box and whisker chart for OAR (grey matter + white matter) and tumour $v_{100}$s comparing Nom Power Nom Plmt (human-defined placements, nominal power, 10000 samples), Reopt Power Nom Plmt (human-defined placements, reoptimized powers, 1000 samples), Nom Power Opt Plmt (SA-optimized placements, nominal power, 10000 samples), and Reopt Power Opt Plmt (SA-optimized placements, reoptimized power, 1000 samples).

Figure 5 shows box and whisker plots of the v100 values of the OARs and the tumour. The first two sets of data, which are labelled Nom Plmt (Nominal Placement) in Fig. 5, present the Gaussian source deviation results discussed above with and without power re-optimization. It is clear that all tumour $v_{100}$s are desirably controlled as observed from the histograms; OAR $v_{100}$ distributions are generally insignificantly affected, except for some outliers, namely T4 and T7.

### Effect of SA position optimization

All observations made so far assume the nominal source locations are planned by a human using clinical heuristics. This section instead analyzes source locations optimized using PDT-SPACE. We generated a set of source locations by running PDT-SPACE with SA placement settings that optimizes for both source locations and powers. The SA placement is constrained to use the same injection surface and the same set of light sources as the original placements. Allowing PDT-SPACE to select the number of light sources can improve treatment plans further^31^; we choose to constrain the number of sources to facilitate easier comparison of uncertainty effects with clinical heuristics vs. SA-optimized source placements. The previously defined procedure for evaluating the impact of source position uncertainty is then applied to the SA-optimized source placements, leading to the last two (green and purple) sets of data in Fig. 5.

We first observe that the nominal $v_{100}$ values for OARs marked in red triangles are reduced in all tumour models in the SA-optimized placements. The prior outliers, T4 and T7, have now improved significantly in both their expected values and spreads, regardless of whether power re-optimization was applied or not. The tumour $v_{100}$ distributions with optimized placements but power values chosen assuming the placement is exact (Nom Power Opt Plmt) have a larger spread of outliers towards the lower end compared to nominal power nominal placement, which is undesirable. But by applying PDT-SPACE power re-optimization on the SA-optimized placements, the same observations apply as we found with human-defined placements — tumour $v_{100}$s are desirably controlled at $98\%$, and OAR $v_{100}$s may be less desirable than without power re-optimization. For both the tumour and OARs, the $v_{100}$ values appear to be generally improved with the SA-optimized placements. Paired t-tests across the nine tumour models were performed to provide statistical support, showing significant increases for Nominal vs Opt+Reopt ($p\approx 0.01$), while OAR differences ($p\approx 0.2$) and Nominal vs Opt tumour differences ($p\approx 0.08$) remain not statistically significant ($p>0.05$).

T9 is the only outlier where the final SA-optimized OAR $v_{100}$ distributions do not match the expected nominal $v_{100}$s. After visualizing the tumour and source placements more closely, we observe that this is related to the tumour shape being irregular. SA optimization would likely perform better if the program were allowed to add or remove light sources, instead of being constrained to the same set of light sources as the human-created placement.

## Discussion

### Discussion regarding power uncertainty

Simulations show that treatment outcomes are minimally affected by source power uncertainty, even under the largest variation permitted by current medical optical fibre manufacturing standards. In practice, additional factors such as fibre-tissue coupling, blood pooling near fibre tips, or local tissue damage may further reduce effective light output. However, even under a conservative worst-case $\pm 20\%$ power variation, PDT-SPACE exhibits limited sensitivity, suggesting that broader uncertainty ranges would not qualitatively alter these conclusions.

PDT-SPACE was modified to account for power uncertainty, with optimization at the minimum source power (Min-only) providing the most reliable performance. This approach reduces tumour $\Delta v_{100}$ from $1.0\%$ to $0.7\%$ in the $\pm 20\%$ case, with proportionally larger reductions at smaller uncertainty levels. Given that power uncertainty produces only minor effects (typically $<1\%$ in tumour $\Delta v_{100}$ and $<0.5\eta$ in OAR metrics), further investigation of more complex strategies was not pursued.

### Discussion regarding position uncertainty

Position uncertainty produces a larger impact on treatment outcomes than power uncertainty. Under Gaussian perturbations, the spread of tumour $v_{100}$ exceeds the worst-case power-uncertainty deviations, with tumour coverage dropping to $\sim 95\%$. Worst-case positional uncertainty produces 2–$3\times$ larger deviations than Gaussian perturbations, reflecting the simultaneous deflection of all sources by up to 3mm. The impact is also direction-dependent, leading to increased variability.

SA-optimized source placements improve placement quality by reducing nominal OAR $v_{100}$ by $36\%$. Source placement optimization alone (Nominal vs. Opt) does not yield statistically significant changes in tumour $v_{100}$ ($p\approx 0.08$), reflecting non-linear behaviour across tumour models. As shown in Fig. 5, smaller tumours with fewer sources (T4, T5, T7, T9) exhibit greater variability, while tumours with more sources ($T1-T3$) are more robust. Additional sources increase the degrees of freedom available to PDT-SPACE during optimization and produce overlapping fluence profiles, reducing sensitivity to individual source displacements.

Accurate knowledge of source placement is therefore important for effective planning. Power re-optimization using measured source positions tightens tumour coverage around $98\%$, with a small increase in OAR $v_{100}$ as a trade-off. Paired t-tests show statistically significant improvements in tumour coverage ($p\approx 0.01$), while OAR differences remain not significant ($p\approx 0.2$). Together, these results indicate that optimized placement combined with online power re-optimization reduces sensitivity to positional uncertainty and limits the need for online dosimetry monitoring.

### General implication

In our prior OP uncertainty study^16,17^, tumour and brain OPs were perturbed with Gaussian noise ($30\%$ standard deviation). Under OP uncertainty, tumour $v_{100}$ varied by only a few percentage points ($-2\%$ to $+3\%$), whereas OAR $v_{100}$ showed much larger deviations (up to 1.5–$1.7\times$ nominal values). In contrast, the present study shows that power uncertainty has minimal impact, and position uncertainty introduces moderate deviations that can largely be mitigated through re-optimization. This comparison indicates that OP uncertainty remains the dominant contributor to variability in treatment outcomes.

At tumour boundaries, reduced fluence overlap from different light sources increases fluence variability, as tissue beyond the tumour edge may receive photons from only a single source. As a result, displacement of a marginal source toward the tumour centre can reduce fluence beyond the edge and increase the risk of under-treating infiltrating tumour cells.

### Future work

This work could be extended to analyze a combination of the uncertainties in light sources with variable OPs^16,17^ and heterogeneous photosensitizer concentration^54^.

## Conclusion

The uncertainties in the application of light sources have been analyzed in two aspects: power delivery uncertainty and source position uncertainty. Simulations on power delivery uncertainty showed little impact on final light delivery outcomes; the impact on tumour light dose coverage can be further reduced by enabling the minimum-only power optimization option in PDT-SPACE. Position uncertainty has shown a greater impact on the final outcome, and the impact is dependent on the direction towards which the light source deviates. Performing a measurement on the light sources after the physical injection and allowing PDT-SPACE to optimize source powers targeting the measured locations can reliably achieve a $98\%$ tumour coverage, with some possibility of increasing OAR damage. Using PDT-SPACE-optimized source placements consistently lowers OAR damage, with slightly more sensitivity to the light sources’ position uncertainty. Measuring source locations and re-optimizing for power allocation reduced the variability in this case as well, and the combination of SA-optimized placements and power re-optimization with measured source locations led to the best average results overall.

## Supplementary Information

Supplementary Information 1.

Supplementary Information 2.

Supplementary Information 3.

## Data Availability

All the FullMonte tools, including FullMonteWeb are available at http://FullMonte.org/. The source code of the tools and all used meshes and datasets in the manuscript are available at https://gitlab.com/FullMonte. All the numerical data obtained in simulations of power and positional uncertainty can be found in the spreadsheets in Supplementary Materials.
