# Supplementary material for: Robustness of interstitial photodynamic therapy treatment planning under power and positional uncertainties in light delivery
Source: Sci Rep. 2026 Mar 5;16:12247. doi: 10.1038/s41598-026-42421-2 (PMC13079899; doi:10.1038/s41598-026-42421-2)
Supplement: Supplementary file 1 — Supplementary Information 1. [file 41598_2026_42421_MOESM1_ESM.pdf]

Robustness of Interstitial Photodynamic Therapy Treatment Planning under Power and Positional Uncertainties in Light Delivery: Supplementary Information

Shuran Wang[1*], Tina Saeidi[2], Lothar Lilge[2,3], and Vaughn Betz[1]

[1]Edward S. Rogers Sr. Department of Electrical and Computer Engineering, University of Toronto, 10 King's College Rd, Toronto, ON M5S3G8, Canada
[2]Department of Medical Biophysics, University of Toronto, 101 College Street, Toronto, ON M5G1L7, Canada
[3]Princess Margaret Cancer Centre, University Health Network, 101 College Street, Toronto, ON M5G1L7, Canada
[*]shuran.wang@mail.utoronto.ca

Tumor Models

The simulated tumor models and their respective source placements are reused from the team's previous work[1,2]. Defined injection constraints are reused from the authors' previous work[3]. Figure S1 shows Paraview[4] visualizations of tumour model shapes in the brain models, showing only the skull and tumour regions.

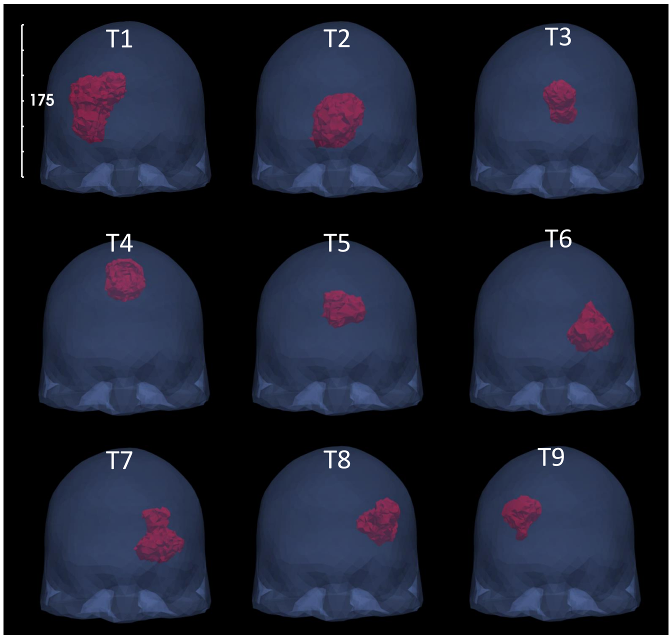

Figure S1. Paraview visualizations of all tumor models viewed from the front of the brain. All meshes are 175 *mm* in height.

Histograms for Medium and Small Tumour Groups

Figures S2 and S3 are the histogram results obtained with the same experimental settings as Figure 3 in the main text.

As the number of sources decreases, we can see the worst case histograms gradually becoming fuzzier, and the expected values approaching the nominal values. The fuzziness is caused by a lack of sample variations since the number of sources is small. For an experiment with 20 sources, the permutation number is 8^{20} as we evaluate 8 directions of source position deviation; for an experiment with 3 sources, the permutation number is $8^3 = 512$, which is much lower than the 10,000 positions sampled, and thus the oversampling of the experiment introduces unnecessary noise in the Worst Case histogram. Gaussian sampling of data eliminates this behaviour and obtains smoother and more accurate distribution curves.

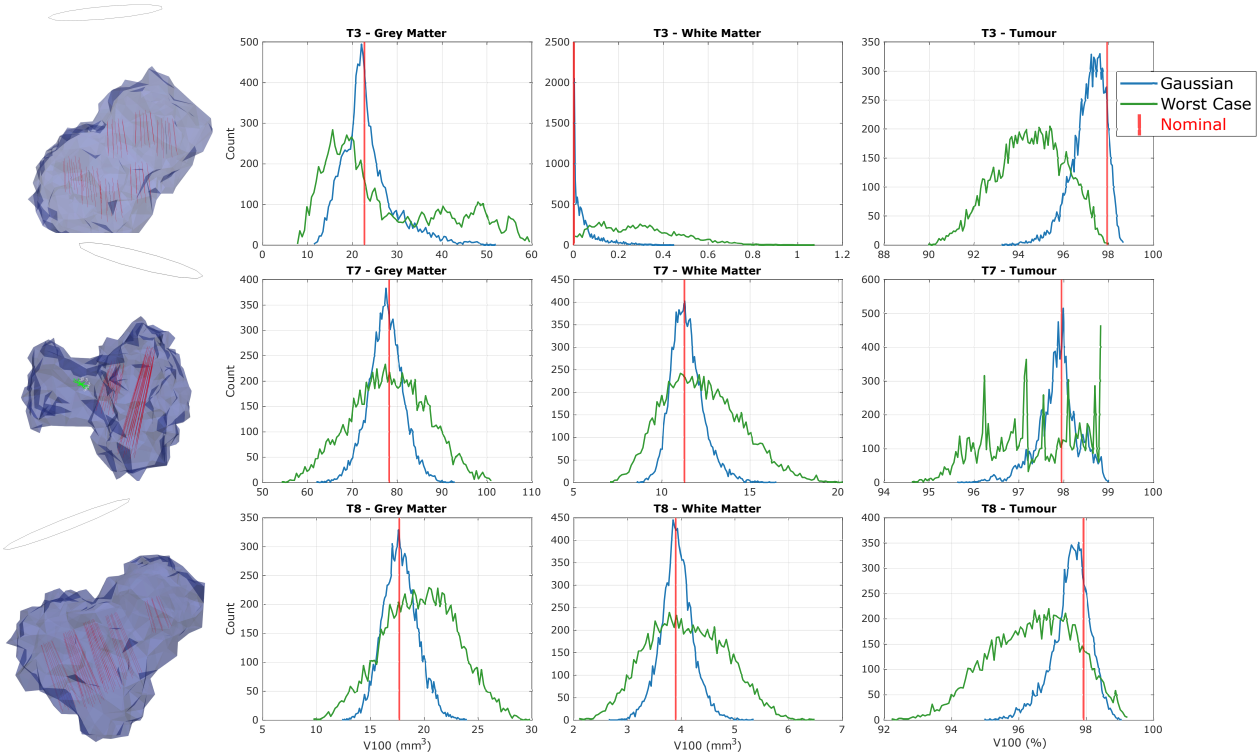

Figure S2. v_{100} histograms under source position uncertainty for the medium 3 tumour models. T3 has 7 line sources; T7 has 6 line sources and 1 point source; T8 has 7 line sources and 2 point sources.

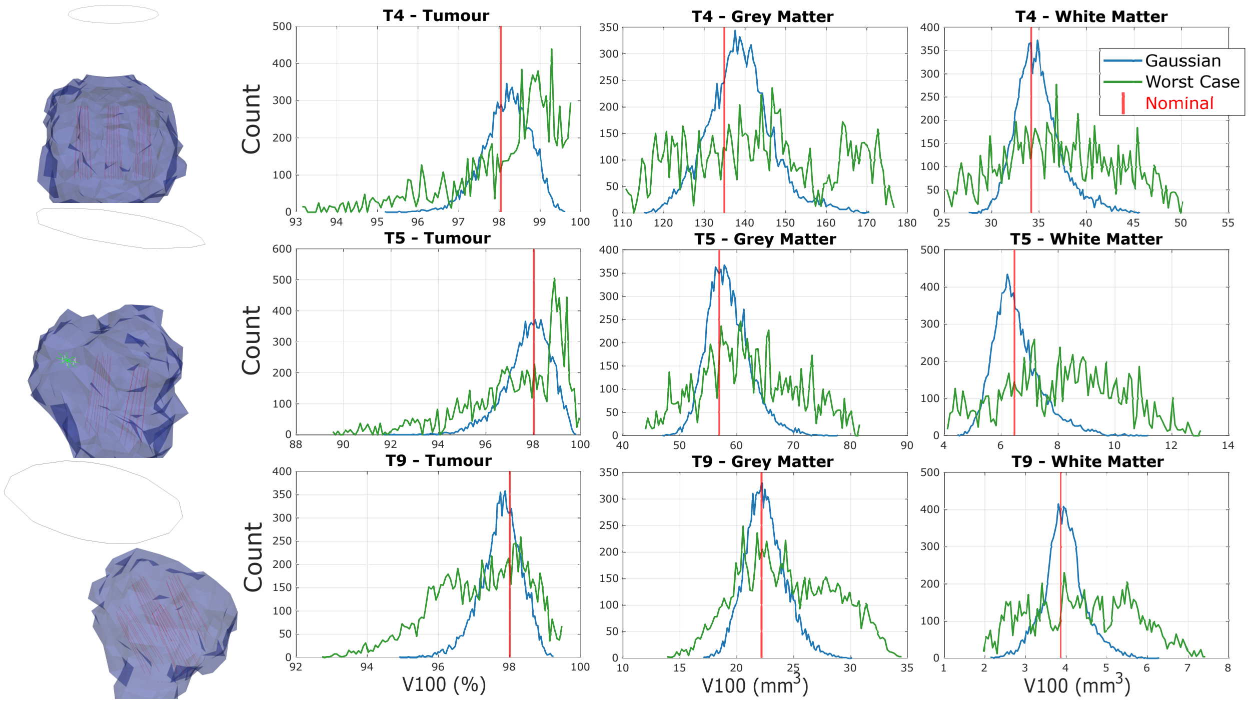

Figure S3. v_{100} histograms under source position uncertainty for the smallest 3 tumour models. T4 has 3 line sources; T5 has 2 line sources and 1 point source; T9 has 4 line sources.

Figures S4 and S5 are the histograms plotted after introducing power re-optimization, under the same experimental settings as Figure 4 in the main text.

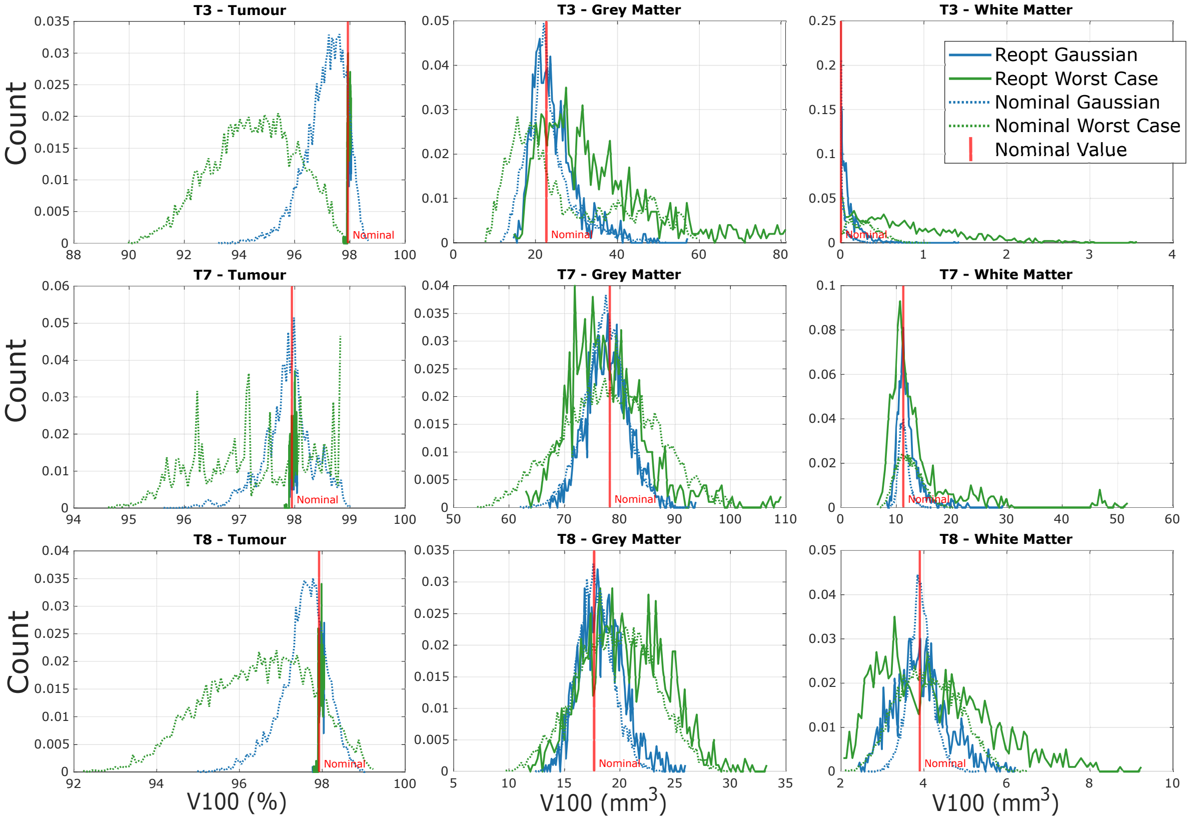

Figure S4. v_{100} histogram comparison between nominal power vs. power re-optimization for the medium 3 tumour models. T3 has 7 line sources; T7 has 6 line sources and 1 point source; T8 has 7 line sources and 2 point sources.

Numerical Simulation Data

All the numerical data obtained in simulations of power uncertainty can be found in the spreadsheet at: https://docs.google.com/spreadsheets/d/1UAniySRVYhnsdc8IJdhGgqJBNL9j58qN7QvtUQcMvzo/edit?usp=sharing.

All the numerical data used to generate the position uncertainty histograms can be found in the spreadsheet at: https://docs.google.com/spreadsheets/d/1GScQ4PAkeByrORX7h-Xr-UbrI_8W4fGEy86g29rXDOo/edit?usp=sharing.

References

1. Yassine, A.-A., Lilge, L. & Betz, V. Optimizing interstitial photodynamic therapy planning with reinforcement learning-based diffuser placement. *IEEE Trans. Biomed. Eng.* **68**, 1668–1679 (2021).

2. Yassine, A.-A., Lilge, L. & Betz, V. Machine learning for real-time optical property recovery in interstitial photodynamic therapy: a stimulation-based study. *Biomed. Opt. Express* **12**, 5401–5422 (2021).

3. Wang, S., Saeidi, T., Lilge, L. & Betz, V. Integrating clinical access limitations into iPDT treatment planning with PDT-SPACE. *Biomed. Opt. Express* **14**, 714–738 (2023).

4. Ahrens, J., Geveci, B. & Law, C. Paraview: An end-user tool for large data visualization. *The visualization handbook* **717** (2005).

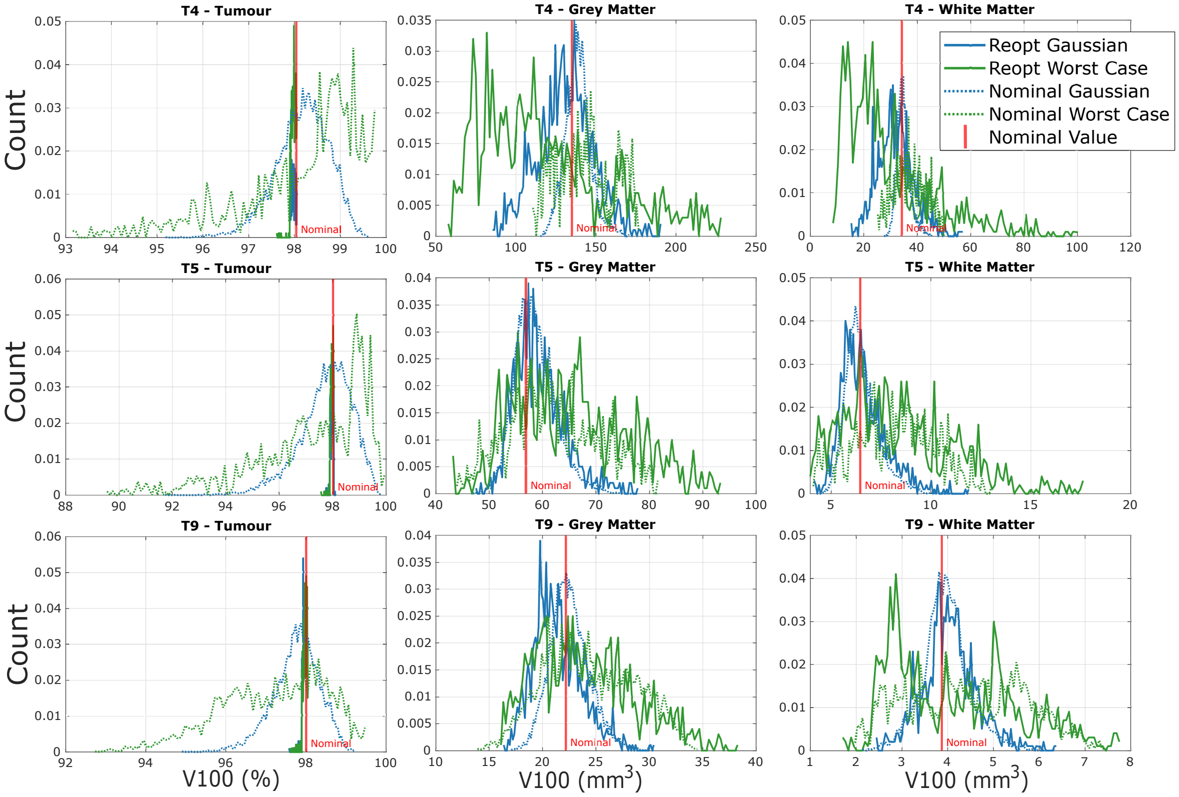

Figure S5. v_{100} histogram comparison between nominal power vs. power re-optimization for the smallest 3 tumour models. T4 has 3 line sources; T5 has 2 line sources and 1 point source; T9 has 4 line sources.
